# Age-Related Changes in Electroencephalographic Signal Complexity

**DOI:** 10.1371/journal.pone.0141995

**Published:** 2015-11-04

**Authors:** Filippo Zappasodi, Laura Marzetti, Elzbieta Olejarczyk, Franca Tecchio, Vittorio Pizzella

**Affiliations:** 1 Dept. of Neuroscience, Imaging and Clinical Sciences, ‘G. d’Annunzio’ University, Chieti, Italy; 2 Institute for Advanced Biomedical Technologies, ‘G. d'Annunzio’ University, Chieti, Italy; 3 Nalecz Institute of Biocybernetics and Biomedical Engineering, Polish Academy of Sciences, Warsaw, Poland; 4 Laboratory of Electrophysiology for Translational neuroScience (LET’S), ISTC, National Research Council (CNR), Rome, Italy; 5 Unit of Imaging, IRCCS San Raffale Pisana, Cassino, Italy; University of Jaén, SPAIN

## Abstract

The study of active and healthy aging is a primary focus for social and neuroscientific communities. Here, we move a step forward in assessing electrophysiological neuronal activity changes in the brain with healthy aging. To this end, electroencephalographic (EEG) resting state activity was acquired in 40 healthy subjects (age 16–85). We evaluated Fractal Dimension (FD) according to the Higuchi algorithm, a measure which quantifies the presence of statistical similarity at different scales in temporal fluctuations of EEG signals. Our results showed that FD increases from age twenty to age fifty and then decreases. The curve that best fits the changes in FD values across age over the whole sample is a parabola, with the vertex located around age fifty. Moreover, FD changes are site specific, with interhemispheric FD asymmetry being pronounced in elderly individuals in the frontal and central regions. The present results indicate that fractal dimension well describes the modulations of brain activity with age. Since fractal dimension has been proposed to be related to the complexity of the signal dynamics, our data demonstrate that the complexity of neuronal electric activity changes across the life span of an individual, with a steady increase during young adulthood and a decrease in the elderly population.

## Introduction

Progressive brain dysfunction in physiological aging is primarily due to a loss of synaptic contacts and abnormal neuronal apoptosis [1]. In healthy elderly individuals, the maintenance of brain activity is promoted by neural and synaptic redundancy, as well as plasticity mechanisms secondary to physical and mental training, these remodel the brain both functionally and structurally [2]. Since age is the main risk factor for neurodegenerative disorders, it is crucial to differentiate between physiological and pathological brain aging by means of techniques that are able to measure and track changes in brain activity. Specifically electromagnetic brain activity, as revealed by electroencephalography (EEG) or magnetoencephalography (MEG), is a convenient non-invasive tool to characterize neural pool functioning and ultimately brain dynamics. Previous neurophysiological assessments by EEG described age related changes of resting state activity of the whole cortical mantle [3–5]. Indeed, progressive neural specialization and global integration of the brain networks during development and maturation, as well as the loss of synaptic connections and neuronal apoptosis in physiological brain aging, also result in a change of dynamics of the electrophysiological data [1].

Brain dynamics reveals intrinsic modulations over time characterized by scale free properties, i.e. the existence of a statistical similarity among details at different temporal scales [6]. The presence of these fluctuations have been observed in several physical systems as the result of complex non-linear interactions at multiple levels, signaling the permanence in a critical state in-between the two extreme conditions of order and randomness [7]. Several models, at different scales, have been proposed to infer the generation of non-linear brain dynamics [8]. Indeed, evidence exists that complex temporal fluctuations of signals detected by electrophysiological techniques reflect nonlinear dynamical processes [9]. Since classical spectral analysis is not able to describe the non-linear dynamics of EEG/MEG signals, non-linear measures have been used to disclose new standpoints for the comprehension of brain functioning [10]. Specifically, an EEG/MEG time series displays fractal properties if statistical similarity can be recovered at different scales in its temporal fluctuations. In this case, scaling properties dictate a scale-free behavior, i.e. the fluctuations at short time scales are related to those at long ones. This behavior is quantified by fractal dimension [7]. Fractal dimension has been previously used to measure the complexity of system dynamics [11, 12], although no general consensus exists that the fractal dimension of EEG signals straightforwardly relates to the complexity of brain dynamics. In this framework, the term “complexity” has been associated to the temporal structure of EEG signals, which usually sit in an intermediate state between two extreme non-physiological situations: absolute absence of variability (e.g. constant signal) and pure unstructured randomness (e.g. white noise). Since the reduction or even the lack of complexity in brain signals has been observed in several neurological disorders and has been associated to dysfunctions in brain activity [13–17], the complexity has been hypothesized to be associated to the efficiency of the brain system [7].

Different algorithms have been proposed to quantify the fractal nature of EEG signals, both at rest and during task [10, 18]. Most of these techniques are based on time-delayed procedures embedding the physiological time series in a multidimensional phase space. Conversely, the approach proposed by Higuchi [19] quantifies fractal dimension directly in time domain, without the necessity of embedding the data in a phase space. Therefore, the algorithm is also less time-consuming and overcomes the problems bound to the choice of the embedding dimension. Moreover, it has been demonstrated that it works even with relatively short epochs. Its applicability to EEG data and high noise-resistant performance has been previously demonstrated both in simulated and in real data [11].

In this work, we aimed at evaluating whether the fractal dimension of EEG activity, as measured by Higuchi's algorithm, is modulated across healthy aging. Indeed, our working hypothesis is that EEG fractal dimension at rest displays a dependence on age, which might yield additional information compared with conventional spectral measures.

## Materials and Methods

Forty healthy volunteers (aged 16–85, including 14 females) were enrolled in this study. All subjects were right-handed, as confirmed by the Edinburgh Manuality test [20], were not receiving any pharmacological treatment at the time of recordings and resulted normal using both neurological examination and brain magnetic resonance imaging. The experimental protocol was approved by the Ethical Committee of "San Giovanni Calibita" hospital, in Isola Tiberina, Rome, and by the Ethical Committee of”G. d’Annunzio” University, in Chieti. All subjects signed a written informed consent.

Five minute open-eyed electroencephalographic recordings were acquired while subjects sat on a comfortable armchair fixating a cross displayed in the center of the screen. EEG activity was recorded by 19 electrodes positioned according to the 10–20 International EEG system (F1, F7, T3, T5, O1, F3, C3, P3, FZ, CZ, PZ, F2, F8, T4, T6, O2, F4, C4 and P4) and referenced to a fronto-central electrode. Additionally, electrooculogram and electrocardiogram data were recorded to monitor for ocular and cardiac artifacts. Data were filtered between 0.1–70 Hz and sampled at 256 Hz.

A semiautomatic procedure based on Independent Component Analysis (ICA) [21] was applied to identify and eliminate artifacts (e.g. eye movements, cardiac activity, scalp muscle contractions). The Reference Electrode Standardization Technique was used to standardize the reference of scalp EEG recordings to a point at infinity that, being far from all possible neural sources, acts like a neutral virtual reference [22, 23].

Fractal dimension (FD) was calculated for each EEG channel by means of the algorithm proposed by Higuchi [19]. The FD of a time series is based on the direct measure of the mean length of the curve L(k), by using a segment of k samples as measure unit, as briefly explained in the following.

From any given time series of N samples: y(1),y(2),…y(N) k new time series are defined as:

$$y_{k}^{m}\text{: y}\left(\text{m}\right),\text{y}\left(\text{m}+\text{k}\right),\text{y}\left(\text{m}+2\text{k}\right),\;\ldots \;y\left(\text{m}+int\left(\frac{N-\text{m}}{\text{k}}\right)\text{ k}\right)$$

where m is the initial time sample and k is the time step. The length L_m_(k) of each curve $y_{k}^{m}$ is calculated as follows:

$$L_{m}^{}\left(\text{k}\right)\;=\;\frac{1}{\text{k}}\left[\frac{N-1}{\text{int}\left(\text{p}\right)\;k}\left(\sum_{\text{i}=1}^{\text{int}\left(p\right)}\left|y\left(\text{m}+\text{i k}\right)-y\left(m+\left(\text{i}-1\right)\text{ k}\right)\right|\right)\right]$$

where p = (N-m)/k.

For each possible k time step, the length of the curve L(k) is evaluated by averaging the k sets of L_m_(k) values, as in:

$$\text{L}\left(\text{k}\right)\;=\;\frac{1}{\text{k}}\sum_{\text{m}=1}^{\text{k}}{\text{L}}_{m}\left(\text{k}\right)$$

The calculation is repeated for k values ranging from 1 to k_*max*_. The curve L(k) is said to have fractal dimension (FD) if:

$$\text{L}\left(\text{k}\right)\sim {\text{k}}^{-\text{FD}}$$

In this case, the plot of log(L(k)) against log(k) should fall on a straight line with slope equal to -FD. Therefore, FD can be obtained by means of a least-squares linear best-fitting procedure.

Fractal dimension increases as the k parameter growths and it reaches a constant value for k > k_max_. The point at which FD plateaus is considered to be the value of k_max_. Fractal dimension is a measure of the complexity of the curve and thus of the time series that this curve represents, ranging from 1 for deterministic constant functions to 2 for a totally stochastic signal, as white noise.

In this work, k_max_ was found to be equal to 16. FD was calculated in windows of 10 second length and averaged over these windows, for each EEG channel. As a global measure of FD, all FD values obtained for the single EEG channels were averaged.

Moreover, Power Spectral Density (PSD) was estimated for each EEG channel via the Welch procedure (4 s epoch, resulting in a frequency resolution of 0.25 Hz; Hanning windowing; 60% overlap, about 70 artifact free trials used for the estimation). A global PSD measure was calculated as the mean of the PSDs obtained by each of the 19 EEG channels.

Since a bulk of evidence in the literature demonstrated that alpha rhythm slows down with age [24–29], band powers were defined according to the individual frequency bands [30]. The individual alpha frequency peak (IAF) was first calculated as the frequency with maximal PSD in the 7–13.5 Hz interval in parieto-occipital electrodes. Thus, the following bands were considered: delta from 1 Hz to 4 Hz; theta from 4.25 Hz to the minimum between 7.5 Hz and IAF-2 Hz; low alpha from IAF-2 Hz to IAF; high alpha from IAF +0.25 Hz to IAF+2 Hz; beta from 13 Hz to 30 Hz; gamma from 30.25 Hz to 45 Hz. Absolute power values were log-transformed, in order to best follow a Gaussian distribution. Relative band power was also calculated as percentage of band power over the power in the whole frequency range (from 1 to 45 Hz).

The degree of symmetry between EEG activity of homologous areas in the right and left hemispheres, Homologous Areas Symmetry (HArS), was obtained for FD values and for the absolute band power in each band, as follows:

$${\text{HArS}}_{\text{i}}=\frac{{\text{X}}_{\text{RH}}-{\text{X}}_{\text{LH}}}{{\text{X}}_{\text{RH}}+{\text{X}}_{\text{LH}}}$$

“X” being either the fractal dimension or the band powers of electrodes pairs in right (RH) and left (LH) hemispheres. Eight different electrode pairs were considered: Fp2 and Fp1; F4 and F3; F8 and F7; C4 and C3; T4 and T3; P4 and P3; T6 and T5; O2 and O1.

### Statistical analysis

As a first step, we divided our population in 3 groups according to age: young adults (10 subjects younger than 25, mean age: 20.6 ± 2.1 years), adults (14 subjects, range 25–66, mean age: 41.1 ± 15.4 years), older adults (16 subjects over 66, mean age: 73.6 ± 4.8 years). Repeated measures ANOVA was performed on FD values with *Electrode* (Fp2, F4, F8, C4, T4, P4, T6, O2, Fz, Cz, Pz, Fp1, F3, F7, C3, T3, P3, T5 and O1) as within-subject factor and *Age* (<25 years of age, 25≤ years of age ≤66, > 66 years of age) as between subject factor, in order to assess topographical changes of FD across age.

Spearman’s or Pearson’s correlations were applied to identify possible changes of FD, band power or inter-hemispheric symmetries with age. Moreover, linear and quadratic best fitting procedures were applied to model FD and FD HArS values dependency over age.

Finally, to evidence a possible relationship between FD and band power, a regression analysis was carried out, with FD values as dependent variable, including all band powers (delta, theta, low alpha, high alpha, beta and gamma) as independent variables.

## Results

**Fig 1:**
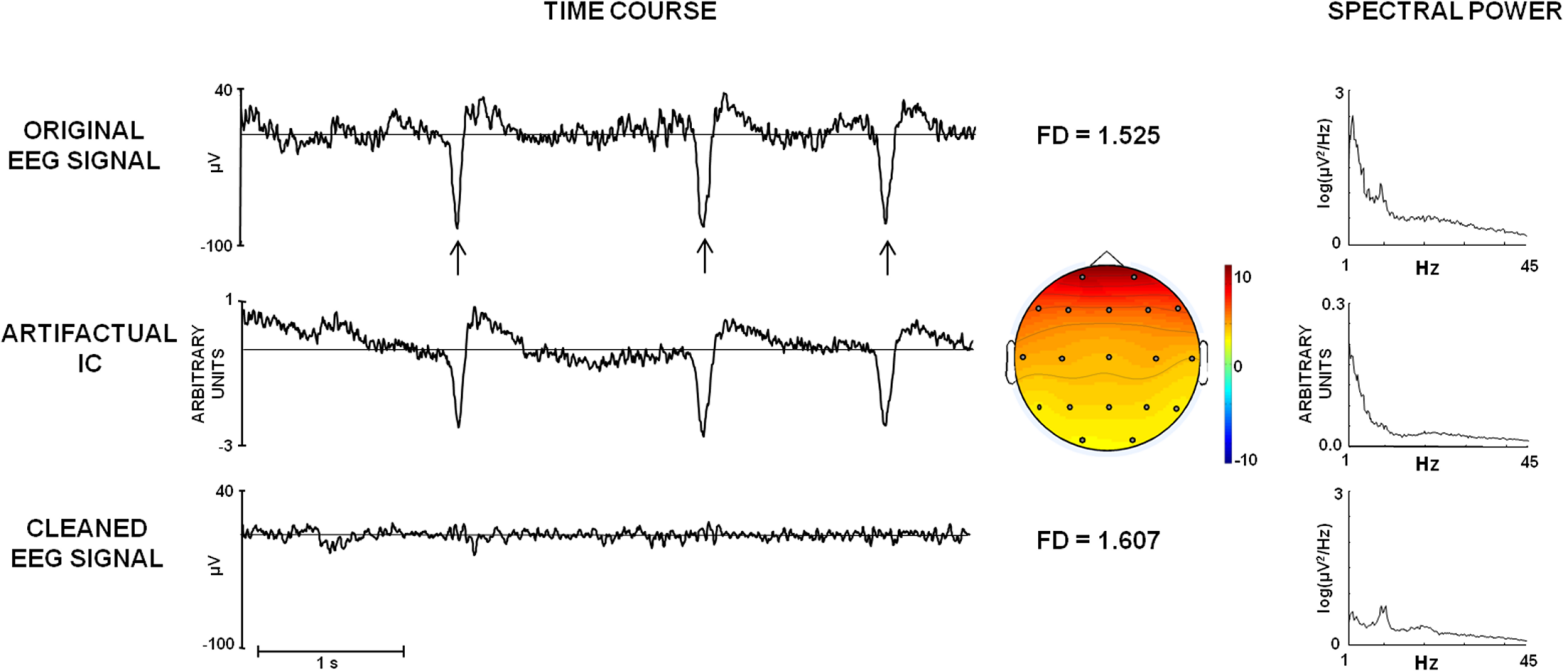
Example of EEG pre-processing by ICA. Time course (5 seconds, filtered between 1 and 70 Hz) and corresponding Power Spectral Density (PSD) of: First row: original EEG signals (Fp2), where the ocular artifacts are evident (vertical arrows), corresponding to the low-frequency activity below the 5 Hz in the PSD. The value of Higuchi’s Fractal Dimension (FD = 1.525) is also displayed. Second row: the estimated IC corresponding to ocular artifacts. We also show the topography of IC weights over the scalp. Third row: EEG signal after removal of the ocular artifactual IC and the FD (1.607). Noteworthy, the ocular artifacts affects FD, being the FD value of the cleaned signal higher than the FD value of the noisy signal.

Fig 1 shows the effect of EEG pre-processing by using ICA. The importance of artifact removal, namely eye blinking in this example, is evidenced by the different FD values found for raw signals (FD = 1.525) and cleaned signals (FD = 1.607). We observed that the presence of ocular and low-frequency artifacts in the data results in lower FD values in comparison to those obtained for cleaned signals. Conversely, muscular contaminations and high-frequency noise result in higher FD values.

**Fig 2:**
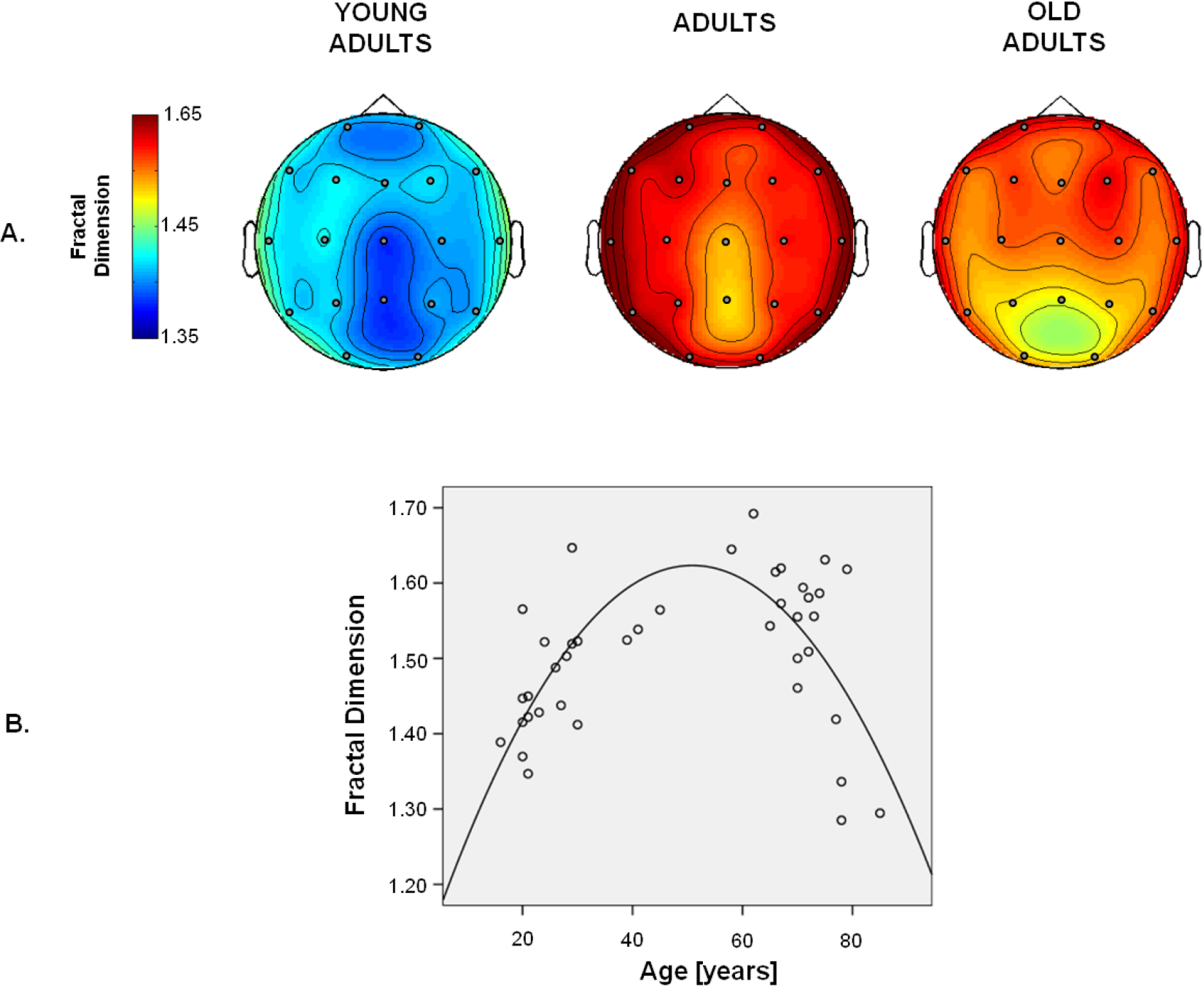
A. Topographies of mean values of fractal dimension in the 3 groups: young adults (<25 years of age), adults (25–66 years of age), old adults (> 66 years of age) B. EEG electrodes are indicated by a full circle. Scatter-plot of fractal dimension values over age (years) and fitting line.

To verify the possible dependence of FD on age, a repeated measures ANOVA was carried out with *Electrode* as within-subject factor and *Age* as between subject factor. The *Age* effect [F(2, 37) = 4.69, p = 0.015, Fig 2A] was found to be significant, indicating a variation of global FD with age. In particular, FD increased from young adulthood (mean ± standard deviation over the whole scalp: 1.41 ± 0.07) to adulthood (1.53 ± 0.08) and decreased in elderly (1.48 ± 0.12). Fractal Dimension age-related changes were not topographically specific, as documented by the absence of the interaction *Electrode* x *Age* (p = 0.822). The *Electrode* effect [F(7.0, 260.3) = 4.22; p<0.001] indicated that FD values significantly differ across EEG sensors. In particular, FD values were higher in frontal and temporal regions with respect to parieto-occipital regions for each age range (Fig 2A).

The plot of the global FD over age (Fig 2B) showed a positive correlation for young adulthood (< 50 yrs; Pearson’s r = 0.59; p = 0.005) and a negative one for old adulthood (> 50 yrs; Pearson’s r = -0.69; p = 0.001). The curve that best fits the data over the whole sample was a parabola (r = 0.67, Fig 2B; F(2, 37) = 15.23; p<0.001).

**Fig 3:**
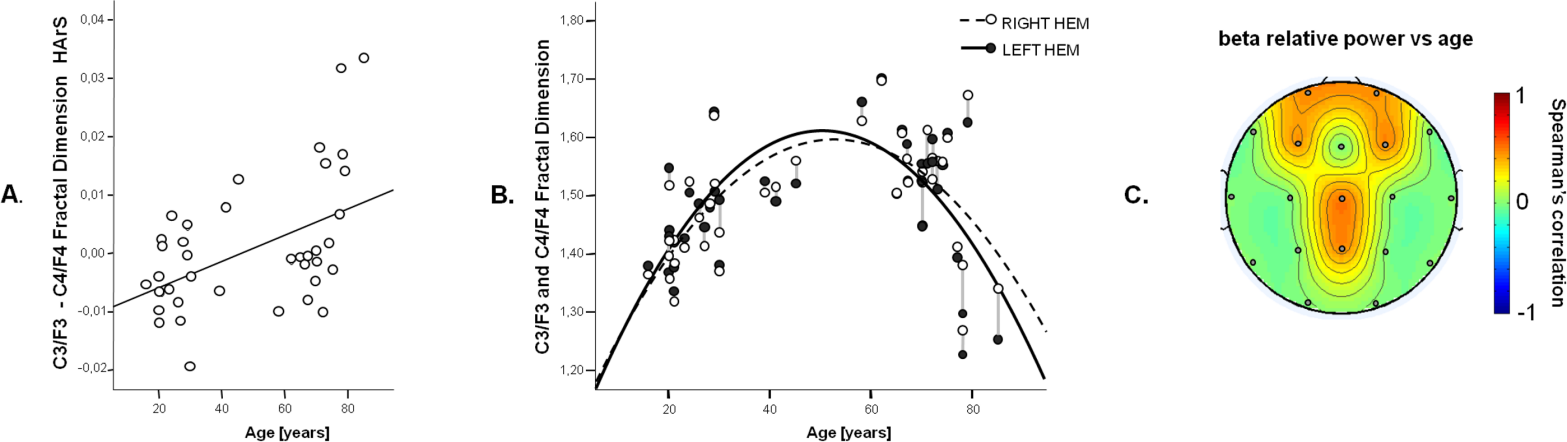
A. Scatter plot of HArS between F3/C3 and F4/C4 over age and fitting line (r = 0.47, F(1,38) = 11.13, p = 0.002) B. Scatter-plot of mean fractal dimension values of F3 and C3 (white circle) and F4 and C4 (black circle) hemisphere over age. The gray lines connect the left and right values of each subject. Fitting line for both values are displayed (dashed line for left hemisphere: r = 0.62, F(2,37) = 11.57, p<0.001; full line for right hemisphere: r = 0.63, F(2,37) = 12.39, p<0.001). C. Topographies of the Spearman’s rho coefficients of the correlation between age and relative power in beta band. Only values for which p <0.05 (FDR corrected) are displayed.

To highlight possible changes of FD inter-hemispheric balance between EEG activities of homologous areas, as measured by HArS, for each couple of homologous electrodes Pearson’s correlation was calculated between FD, HArS values and age (Table 1). A positive correlation was found for the pairs F3/F4 and C3/C4 (Fig 3A), indicating a more pronounced asymmetry in elderly individuals. In particular, this increased asymmetry is due to FD more rapidly decreasing with age in the left hemisphere with respect to the right one (Fig 3B).

**Table 1:** Correlation between interhemispheric symmetry indexes of FD, Theta, Beta and Gamma band Power values between homologous electrodes, and the age.

	Fractal Dimension	Theta band Power	Beta band Power	Gamma band Power
Fp1-Fp2	-0.14 (n.s.)	-0.06 (n.s.)	-0.09 (n.s.)	-0.07 (n.s.)
F7-F8	-0.01 (n.s.)	-0.09 (n.s.)	0.17 (n.s.)	-0.19 (n.s.)
***F3-F4***	***0*.*39 (0*.*012)***	-0.03 (n.s.)	*0*.*27 (n*.*s*.*)*	0.28 (n.s.)
***C3-C4***	***0*.*41 (0*.*008)***	***0*.*34 (0*.*031)***	***0*.*37 (0*.*018)***	***0*.*35 (0*.*028)***
T3-T4	0.10 (n.s.)	0.26 (n.s.)	0.13 (n.s.)	0.27 (n.s.)
T5-T6	0.21 (n.s.)	-0.06 (n.s.)	0.11 (n.s.)	0.07 (n.s.)
P3-P4	0.25 (n.s.)	0.14 (n.s.)	0.09 (n.s.)	0.17 (n.s.)
O1-O2	-0.02 (n.s.)	-0.19 (n.s.)	-0.14 (n.s.)	-0.12 (n.s.)

Pearson’s r (p-value; n.s.: p>0.05)

### Relationship between spectral characteristics and age

A dependence of delta, theta and low and high alpha band powers on age was found. The curve best fitting these band power values over age was a parabola (consistently r >0.39 for the different bands). Considering the HArS power values, band power asymmetry between F3/C3 and F4/C4 positively correlated with age, in theta (r = 0.41, p = 0.008), as well as at high frequencies (beta: r = 0.33, p = 0.038; gamma: 0.35, p = 0.025, Table 1). This asymmetry is due to a higher power in the right hemisphere than in the left for the older subjects (paired sample t-test between F3/C3 and F4/C4 power values: theta t(15) = 2.68, p = 0.017; beta t(15) = 2.39, p = 0.030; gamma t(15) = 2.54, p = 0.022).

No relationship was found between relative power values and age, except for the beta band in fronto-central areas (Fig 3C).

### Relationship between fractal dimension and spectral characteristics

Global power over the whole scalp negatively correlated with global FD values in lower frequencies: delta (Pearson’s r = -0.65, p<0.001), theta (r = -0.61, p<0.001), low alpha (r = -0.71, p<0.001) and high alpha (r = -0.63, p<0.001). This correlation, when resolved for each electrode, was not topographically specific, as it was found over all EEG electrodes (consistently p <0.05, FDR corrected). Relative power values did not correlate with FD values.

To further investigate the relationship between band powers and FD values, a regression analysis was performed, with FD as the dependent variable. All global band powers over the whole scalp (delta, theta, low alpha, high alpha, beta and gamma) were included as independent variables. Only delta, low and high alpha and beta bands entered the model:

FD=1.85–0.09lowalpha+0.21beta–0.13highalpha–0.11delta

The sign of the model coefficients tells us that a decrease of FD is associated with an increase of power in the lower frequencies (low and high alpha, delta) and to a decrease of power in the higher frequencies (beta). The 92% of FD variance was explained by this model, with, in particular, 50% accounted for by low alpha (F(1,38) = 38.48, p<0.001), adjunctive 36% by beta (F(2,37) = 113.87, p<0.001), and only adjunctive 3% by high alpha (F(3,36) = 97.92, p<0.001) and adjunctive 3% by delta (F(4,35) = 104.66, p<0.001).

## Discussion

In this work, the dependence on age of the complexity of the EEG dynamics has been evaluated by means of fractal dimension. Our data show that in the healthy population fractal dimension of EEG signals follows a U-shape over age, increasing from late-adolescence (16–20 years of age) to adulthood and decreasing in old age. Complexity in neural systems has been associated to functional advantages, as for example a proper dynamic range, a high flexibility and a fast adaptability to environment and an efficient itinerancy across phase space of the dynamics of the system [31]. According to these results, the lower fractal dimension in old age could be the expression of a reduced neural efficiency, due to the age-related degeneration phenomena, such as degradation in neurotransmission [32], compromised structural integrity of the grey and white matter [33, 34] and reduced functional connectivity between networks [35]. Along this line, fMRI studies showed that BOLD variability is lower in older and poorer performing adults [36]. Moreover, a complexity reduction of the EEG dynamics has been paired to a functional impairment in neurological pathologies, such as Alzheimer disease [13–15, 37], or to clinical scores in stroke patients [16].

Golderberg et al. [7, 38] evidenced fractal dynamics involved in physiologic control and described an age-related reduction of complexity in the dynamics of heart rate variability and human gait; however, previous studies that evaluated the EEG complexity during healthy aging reported contradictory results. Some studies found an increased complexity in healthy elderly individuals [39, 40], particularly in the frontal region, while other evidenced a decreased complexity [41]. This discrepancy is possibly due to the difference in the algorithms used to evaluate the complexity of the dynamics. Indeed, all studies on band power modulations found a reduced alpha reactivity, especially in frontal regions, probably coupled to an increased relative power in higher frequencies, corresponding to the beta range. Moreover, network indices derived from graph theory evidenced a shift towards a more random topology in elderly individuals, especially in the beta band [41]. Our data confirmed at rest an increased relative power in the beta band in centro-frontal regions, in accordance with previous results [24, 29, 42–44]. In the elderly group, a shift from posterior to anterior involvement of metabolic responses, as revealed by Positron Emission Tomography (PET) has been documented, resulting in a lower regional cerebral blood flow in posterior regions and a higher flow in the anterior regions compared to young subjects [45].

Our findings confirm the results found by McIntosh et al. [46]. In their paper, McIntosh and colleagues found a spatiotemporal dependence of brain complexity dynamics of electrophysiological signals (EEG and MEG) on aging during a visual perception and a multisensory task. In particular, aging was found to be paired both to a decrease of long-range interaction, predominantly involving cross-hemispheric communication, and to a more pronounced local processing. Our data on electrophysiological activity at rest confirmed a global loss of complexity in the elderly, indicated by an unspecific reduced fractal dimension over the whole scalp, paired to an inter-hemispheric unbalance of the activity from homologous primary motor/premotor areas. Indeed, inter-hemispheric balance between homologous sensorimotor areas plays a crucial functional role. In unilateral stroke, sensorimotor disabilities and worse clinical recovery have been linked to inter-hemispheric unbalance of centro-parietal EEG/MEG activity [47–49]. Moreover, age-related hemispheric asymmetry reductions of homologous areas have been observed in PET studies, and have been interpreted as a compensatory function [45, 50].

In this work, we found that in healthy elderly individuals, inter-hemispheric unbalance of EEG activity takes place. In primary motor and premotor areas, the complexity reduction is faster in the left than in the right hemisphere, resulting in an increased FD inter-hemispheric asymmetry. A slower age-related decrease of complexity in the right hemisphere could be a sign of compensative phenomena occurring in the old brain. Indeed, a possible neural network, including the parietal and frontal areas of the right hemisphere, has been proposed to contribute to cognitive reserve, protecting the brain activity from dysfunction due to age-related changes or diseases [51].

Our data document that in healthy elderly individuals the inter-hemispheric complexity asymmetry is paired to an inter-hemispheric power asymmetry in theta, beta and gamma bands, resulting from increased values in the right versus left hemisphere. To be noted, these two phenomena (fractal dimension asymmetry and band power asymmetry) are probably independent, as the regression model with fractal dimension values as dependent variable and all band powers as independent variables showed that the most variance of fractal dimension was explained by alpha power (50%, negative relationship) and by beta power (36%, positive relationship), but not by theta and gamma power. The theta band in motor areas could account for a role of slower frequency coherent oscillations in mediating the neural representations of hand kinematics [52]. Moreover, although the functional role of oscillations in beta band is not yet well understood [53], beta activity has been traditionally associated to motor processing [54–56], sensorimotor control [57] and cortico-spinal coupling [58]. Gamma band oscillations have been associated to dynamic interactions making efficacious sensorimotor loops [59], attention to stimuli [60], visual search [61] and object recognition [62]. Moreover, it drives cognitive processing arising in widespread neural networks, as well as taking place locally in somatosensory areas, clearly enhancing perception acuity [63–66]. Thus, given the functional role of theta, beta and gamma band oscillations in motor cortices, we can speculate that the changes of inter-hemispheric unbalance in elderly individuals could be related to the functional decline of motor processes, such as motor speed and movement coordination. Indeed, band power of the activity from portions of the cortex, as revealed by extra-cephalic electrophysiological recordings, reflects the simultaneous activity of neurons synchronously firing [67]. Since it is known that approximately 10% of all neocortical neurons are lost over the life span [68], the increase of local band power can be explained by an increase of synchronization of neuronal activity in a specific frequency range. One possible cause of increased synchronous firing activity can be an alteration of the intra-cortical inhibitory structures. Cottone et al. [69] found a right versus left interhemispheric asymmetry increase over age of total power of MEG activity from primary somatosensory cortex of areas devoted to hand control. The authors interpreted these interhemispheric age-related unbalances were due to an increased excitability within the right thalamocortical circuit, impacting left versus right unbalances of spontaneous firing rates and of local inhibitory-excitatory networks.

To quantitatively evaluate the interdependences between the primary motor areas of the two hemispheres, Transcranial Magnetic Stimulation (TMS) protocols can be applied. Indeed, the inhibition via the transcallosal pathway of one hemisphere over the other can be assessed in an indirect way, by the ipsilateral silent period, or in a more direct way, by the modulations of the interhemispheric paired-pulse motor evoked potential [4]. TMS studies reported that interhemispheric inhibition phenomena were asymmetric, with the left hemisphere inhibiting the right more than the right hemisphere inhibiting the left, in right-handed subjects [70, 71].

Signs of less efficiency in contralateral inhibition have been found in old healthy adults compared to younger adults [72, 73]. Since the local inhibitory structures are the target of the transcallosal fiber projections, we can hypothesize that the age-related theta, beta and gamma band power unbalance can be caused by a loss of efficacy of the local inhibitory structures in the elderly.

While a decrease of fractal dimension was found in healthy aging, our observation reported also an increase of fractal dimension in young adulthood, between 20 and 40 years of age. This increase can be the expression of the maturation processes still occurring in this age range. Indeed, EEG studies demonstrated that intermingled delta and theta activities of medium voltage, moderate signs of immaturity, are present in the EEG of awake subjects over posterior regions until the age of 30 [74].

Fractal dimension was found to be bound, independently of age, to an alpha power decrease and beta power increase. Since Fractal Dimension is a mathematical measure of self-similarity in terms of temporal dynamics, the fact that it is differently correlated to different band powers suggests different degrees of long range correlation in dependence on the spectral range of the EEG signal. A multifractal behavior of EEG signals could be hypothesized, as pointed out by Weiss et al. [75]. Further studies are needed to disambiguate the fractal or multifractal nature of EEG signals, as well as to quantitatively compare Higuchi’s fractal dimension with direct measures of long-range correlation in different frequency bands.

In conclusion, we conceive that fractal dimension is a proper measure to track changes of complexity of the dynamics of neuronal activity during ageing. Furthermore, our investigation supports the suitability of Highuchi’s fractal dimension as a proper measure to monitor the maturation and decline of brain organization along life-span, since it yields additional information compared with conventional spectral measures.

## Supporting Information

S1 File Relevant data collection. Sheet 1: Global values of Fractal Dimension (FD) and FD values for each EEG electrode. Sheet 2: Global values of band power and band power values for each EEG electrode.
(XLSX)
